# An experimental investigation and flow-system simulation about the influencing of silica–magnesium oxide nano-mixture on enhancing the rheological properties of Iraqi crude oil

**DOI:** 10.1038/s41598-024-56722-x

**Published:** 2024-03-14

**Authors:** Salem Jawad Alhamd, Mehrdad Manteghian, Amir Hossein Saeedi Dehaghani, Farhan Lafta Rashid

**Affiliations:** 1 Department of Petroleum Engineering, Faculty of Chemical Engineering, Tarbiat Modares University, P.O. Box 14115-111, Tehran, Iran; 2 Department of Petroleum Engineering, College of Engineering, Kerbala University, Kerbala, 56001 Iraq

**Keywords:** Silica–magnesium oxide nanoparticles, Crude oil, Viscosity reduction, Bingham model, Yield stress, Energy science and technology, Engineering, Nanoscience and technology

## Abstract

This study aims to investigate the effects of introducing a 50/50 mixture of silica and magnesium oxide nanoparticles (SNP + MgONP) to the viscosity of Al-Ahdab crude oil (Iraq) at varied concentrations and temperatures. It is observed that the viscosity value drops from 38.49 to 7.8 cP. The highest degree of viscosity reduction is measured to be 56.91% at the maximum temperature of 50 °C and the greatest concentration of 0.4 wt% SM4. The Bingham model can be used to classify the behavior of the crude oil before the Nano-mixture is added. The liquid behavior grew closer to Newtonian behavior once the Nano-mixture was added. Along with a rise in plastic and effective viscosity values, the yield stress value decreases as the concentration of the Nano-mixture increases. The numerical data demonstrate that when the volume proportion of nanoparticles increases, the pressure distribution decreases. Furthermore, as the nanoparticle volume fraction increases, the drag decrease would also increase. SM4 obtains a maximum drag reduction of 53.17%. It is discovered that the sample SM4 has a maximum flow rate increase of 2.408%. Because they reduce the viscosity of crude oil, nanoparticles also reduce the friction factor ratio.

## Introduction

Economically, the petroleum industries face many challenges and obstacles that need quick and inexpensive solutions, the most important of which are transportation operations problems^1^. Pumping power losses happen due to friction forces between the fluids and the oil pipe's wall, and the presence of turbulence flow during oil delivery via the pipeline^2^. Turbulence causes a large amount of energy to be expended in its creation, leaving less energy available to support fluid movement. The distance between the wellhead and the gas–liquid separator in multiphase oil and gas production systems is normally between 10 and 30 kms, although it may be as far as 100 km^3^.

To generate the desired product, crude oil must be transported under added pressure at a sufficiently high pressure. In ordinary petroleum pipeline systems, the amount of pressure applied is typically constrained by design restrictions. As a result, the difficulties brought on by decreasing pressure are magnified when fluids are transported across large distances. Such problems in transportation methods call for more expensive operational costs and larger equipment^4,5^.

Numerous studies were examined approaches to improve crude oil pipeline throughput by lowering drag, heating, and viscosity-breaking^6,7^. Nanomaterials, additives, are a smart and innovative solution. Adding a small quantity of these additives to the fluid can increase pipeline capacity without changing its conditions, lowering friction and turbulence drag and energy needed to pump the fluid^8,9^.

Kumar et al.^10^ summarized nanotechnology, including nanomaterial manufacturing and properties. Nanoparticle applications in oil and gas are given to show cement nanomaterial uses. The authors explained how Nanoparticles affect cement density, viscosity, fluid loss, thickening time, and mechanical characteristics. These mechanisms impact cement's properties when Nanoparticles are introduced. SiO_2_, MgO, TiO_2_, Fe_2_O_3_, Al_2_O_3_, and graphene oxide have been added to cement to improve its properties.

Al-Shargabi et al.^11^ explored nanotechnology applications, Nanoparticle manufacturing, classification, and its various uses in drilling engineering, notably in drilling fluids. Al-Shargabi et al.^11^ explored oil or water-based drilling fluid applications. The nanoparticles utilized have improved many drilling methods. Drilling fluids using silica and alumina nanoparticles have improved rheology, filtration, and thermal stability.

Yakasai et al.^12^ detailed IONP properties, preparation, and usage in oil recovery. The basic IONPs' structural, physicochemical, and physical properties were shown. Followed by iron oxide Nano-fluid (IONF) production. Petroleum industry utilization of IONPs was also discussed. The surface coating efficiency and IONPs' EOR efficacy were compared to other Nanoparticles.

Maleki et al.^13^ tested six injection solutions on light and heavy oil recovery in two formation water brines using a homogenous glass micromodel. All injection solutions except one without additions were made using 40,000 ppm NaCl synthetic seawater (SSW). Other injection solutions were created by dispersing surfactant (CTAB), polyacrylamide (PAM), Nanocomposite silica-based polyacrylamide (NCSP), and Nanocomposite alumina-based polyacrylamide. Visual data revealed that modulating mobility ratio increases sweeping efficiency and oil recovery, while silica and alumina nanocomposite reduce interfacial tension and wettability change.

Rattanaudom et al.^14^ employed a binary surfactant system with hydrophobic and hydrophilic SiO_2_ Nanoparticles (NPs) at a volume ratio of 0.6:0.4. A comprehensive set of experiments was conducted in API brine with an 8:2 weight ratio of NaCl:CaCl_2_ to study micro-emulsion phase behavior, interfacial tension (IFT), surfactant adsorption onto NP surfaces, solution and micro-emulsion viscosity, and oil recovery. NP concentrations and surface attributes affected micro-emulsion phase behavior, ideal salinity (S*), and IFT. Hydrophobic or hydrophilic NPs at 500 ppm can create Type III micro-emulsion and lower S* from 2 to 1.75 wt  in the binary surfactant system. When NP concentration reached 1000 ppm, S* dropped to 1.5 wt%.

This research presents experimental and computational studies with the goal of examining the impact of Nanoparticles on the reduction of viscosity at low temperatures. By reducing its viscosity, this study would aid in assessing the potential of using Nanoparticles to enhance the rheological features of crude oil, which is especially useful during storage and pipeline transport.

## Theory

### Crude oil rheology

Newton's Law of Viscosity defines how mechanical stress affects a fluid's shear stress and shear rate^15^. Despite not following Newton's law, non-Newtonian fluids' viscosity—the ratio of shear stress to shear rate—can fluctuate with shear rate. A fluid is Newtonian if its viscosity does not vary with shear strain, deformation, or rate. Non-Newtonian fluids are still fluids even if their viscosity varies with shear deformation, strain, or rate. Newton's viscosity law^16^:

1 $$ {\varvec{\tau}} \alpha \frac{du}{{dy}} $$

2 $$ {\varvec{\tau}} = \mu \frac{du}{{dy}} $$

$\tau$ shear stress = F/A, µ = dynamic viscosity, and $\frac{du}{{dy}} =$ shear deformation rate. According to Newton's law of viscosity, dynamic viscosity is the coefficient of viscosity.

### Power law model

A value closer to zero suggests more shear-thinningerial. The most common non-Newtonian behavior is shear thinning or pseudoplastic flow. As shear rate increases, fluid viscosity decreases^17^. Suspensions and polymer-structured materials often thin under shear, although other materials may thicken. Oswald de Ville modified an equation for pseudoplastic fluids like crude oil with high resin and asphaltene contents^18^. Thus, crude oil might be modeled as pseudoplastic,

3 $$ \tau = K \gamma^{n} $$

4 $$ \eta_{eff} = K \gamma^{n - 1} $$

K = coefficient of viscosity, **γ** = Shear rate, $\eta_{eff}$. = Effective Viscosity, and n = flow behaviour index.

It may be used to characterise any material that exhibits power law behaviour, which is the proportionate response of stresto shear rate (or a linear plot of viscosity vs shear rate). Power w behaviour is characterised by a linear relationship between viscosity and shear rate. The technical range of the flow behaviour indexn a power law distribution is 0 < n < 1. When ‘n’ is more than one, it indicates that the sample is shear thickening. However, when ‘n’ is less than one, it indicates that the sample is shear thinned. Furthermore, when ‘n’ is equal to one, it indicates that the sample behaves in a Newtonian and viscous manner^19^.

**Figure 1:**
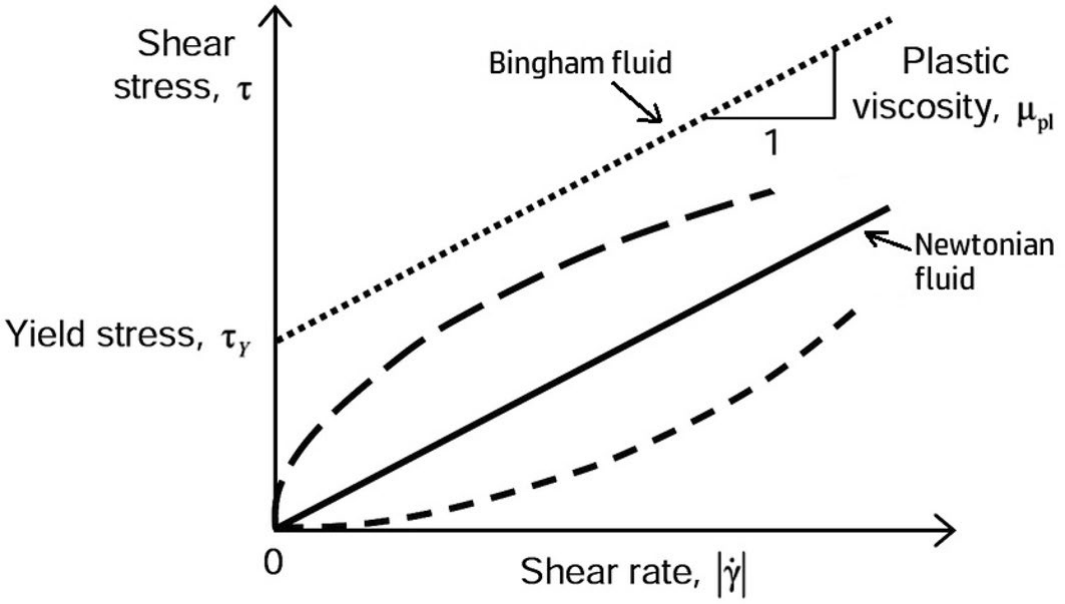
Relationship between fluid shear stress and rate^21^.

The various classes of fluids are shown in Fig. 1 according to the connection that exists between shear stress and shear rate^20^.

### Bingham plastic model

Oil follows the Bingham plastic model, the most common drilling rheology. Without motion, oil and other Bingham plastic fluids resist. This model is constrained by plastic viscosity and yield stress^22^. The fluid resists flowing until the shear stress exceeds a threshold (Fig. 1). Shear stress and rate are linear when fluid flows. The numerical Bingham plastic model specifies flow characteristics as follows^23–25^:

5 $$ \tau = \mu^{\infty } \gamma + \tau_{y} $$

$\mu^{\infty }$ the high shear limiting (or plastic) viscosity, and $\tau_{y}$. is the yield stress. The equivalent viscosity is often referred to as effective or apparent viscosity^26^.

6 $$ \mu_{eff} = \mu^{\infty } + \frac{{\tau_{y} \cdot d}}{6 \cdot v} $$

### Nanoparticle alteration of viscosity

Nanoparticles' catalytic properties reduce viscosity at high temperatures. However, crude oil and Nanoparticles may interact molecularly at low temperatures^27^. Nanotechnology has shown that adding Nano-additives to base oils can minimize friction and wear due to their outstanding lubricating properties. Nano-additives in oil formulations will help the industry meet fuel consumption objectives^28^.

### Tribological mechanism of nanoparticles

While a few metal oxides exhibit the tribological feature, the tribological characteristics of particularly metal oxide Nanoparticles are in part controlled by their structural traits, especially their crystallinity. In contrast to Nanoparticles with inadequate crystallinity. The frictional characteristics of Nanoparticles with imperfectly crystalline structures with comparable average diameters (150 nm) were compared^29,30^.

**Figure 2:**
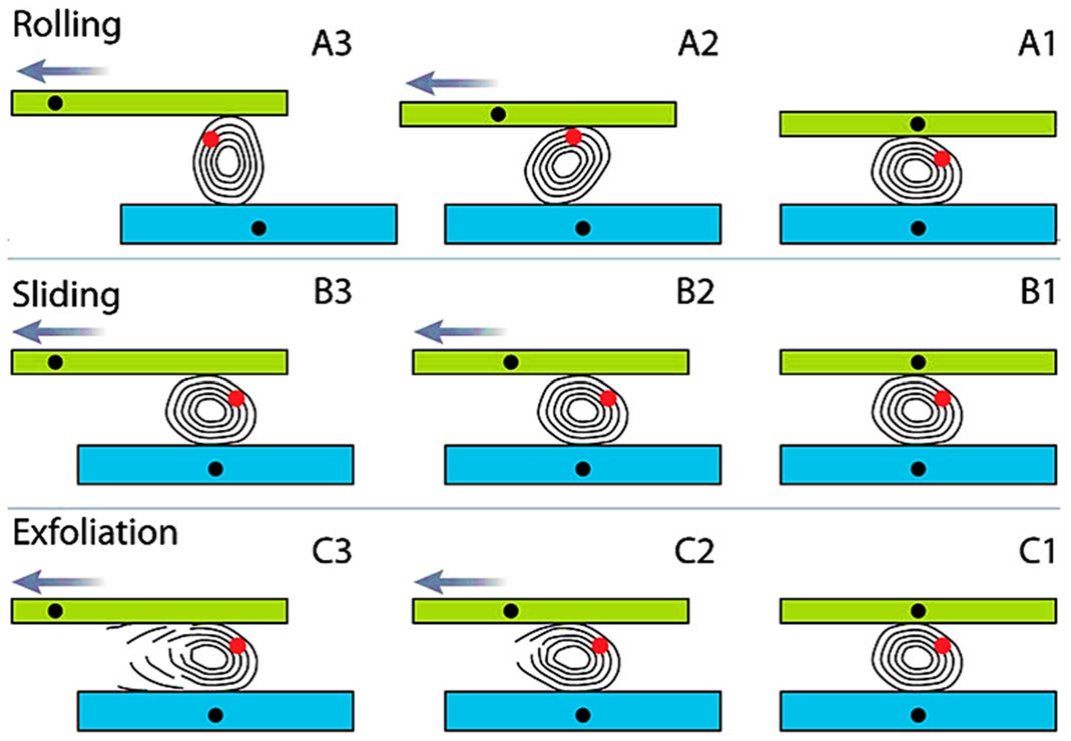
The three major friction processes of multilayered NP are rolling, sliding, and exfoliation^31^.

Rolling, sliding, and exfoliation-transfer (third-body)^31^ are the three fundamental frictional processes that might explain this feature as presented in Fig. 2.

## Materials and procedures

### Materials

This research used crude oil from the Al-Ahdab oil field in Wasit City, central Iraq (API = 25). The crude oil parameters are given in Table 1. This study used silicon dioxide Nano-powder (Hydrophobic) (Hongwu International Group) and Nano magnesium oxide, MgO (Sky Spring Nanomaterials, Inc.) in different concentrations SM1, SM2, SM3, and SM4 (0.1, 0.2, 0.3, and 0.4 wt%), receptively. Nanomaterial parameters are also listed in Table 2. All viscosity tests were conducted at 20, 30, 40, and 50 °C of temperature.

**Table 1:** Specifications of crude oil supplied from Al-Ahdab oil field.

Specifications	Value	Specifications	Value
API gravity	25.74	Salt content mg/l	81.5
Sp.gr at 15 °C (60°F)	0.8999	Water & Sediments %VOL	0.1
Density at 15 °C (60°F)	0.8991	Asphaltenes content (wt%)	1.9
Dynamic viscosity (cP) at 20 °C	38.49

**Table 2:** Specifications of Nanomaterials.

Specification		Specification	
Name	Silicon Dioxide Nanopowder (Hydrophobic)	Name	Nano Magnesium Oxide, MgO
Manufacturer Company	Hongwu International Group	Manufacturer Company	Sky Spring Nanomaterials, Inc
Country	China	Country	USA
Purity	99.8%	Purity	99.9%
Shape	Spherical	Appearance	white powder
APS	20–40 nm	APS	10–40 nm

### Methodology

The fundamental problem in creating Nano-fluids from crude oil is nanomaterial aggregation. This was addressed by increasing nanomaterial uniformity and dispersion. Nanomaterials and oil samples were created separately during mixing. The allowed weight ratios for Nanomaterials to crude oil were 0.1, 0.2, 0.3, and 0.4 wt% silica and magnesium oxide Nanoparticles at an equal ratio of 50/50. Quantities to blend with crude oil samples were prepared using a sensitive balance. To guarantee nanomaterial dispersion, each sample was stirred with a magnetic stirrer for one hour at room temperature after mixing. To achieve a stable, homogenous mixture with minimal agglomeration and great dispersion, a Grant digital ultrasonic bath (XUB Series, U.K.) was utilized for sonication from 30 min to 1 h.

The Brookfield Viscometer, Rheometer, and Texture Analyser (Model DV3TLVKJ0, TKV. 1.0, Spindle V.1.0, USA) measured crude oil-based Nano-fluids' viscosity and Newtonian and non-Newtonian behavior. Viscosity, Shear Stress, and Shear Rate were assessed before and after nanomaterial addition with different concentrations, temperatures, rotation speeds, and momentum for each sample. The temperatures were specified at 20, 30, 40, and 50 °C. Viscosity measurements were based on the rotating viscometer's mechanism. Therefore, the driving torque values needed to rotate a spindle in bulk Nano-fluid were observed. The viscosity measurement device was connected to a bath of flowing water to maintain the appropriate temperature of the parent and crude oil-based Nano-fluids. DVR was determined using the following Eq. ^32^:

7 $$ DVR = \frac{{\mu_{ref - } \mu_{treared} }}{{\mu_{treared} }}*100\% $$

µ_ref_ is the reference viscosity, and µ_treated_ is the viscosity after treatment by Nano-additives.

### Characterisation of nanomaterials

**Figure 3:**
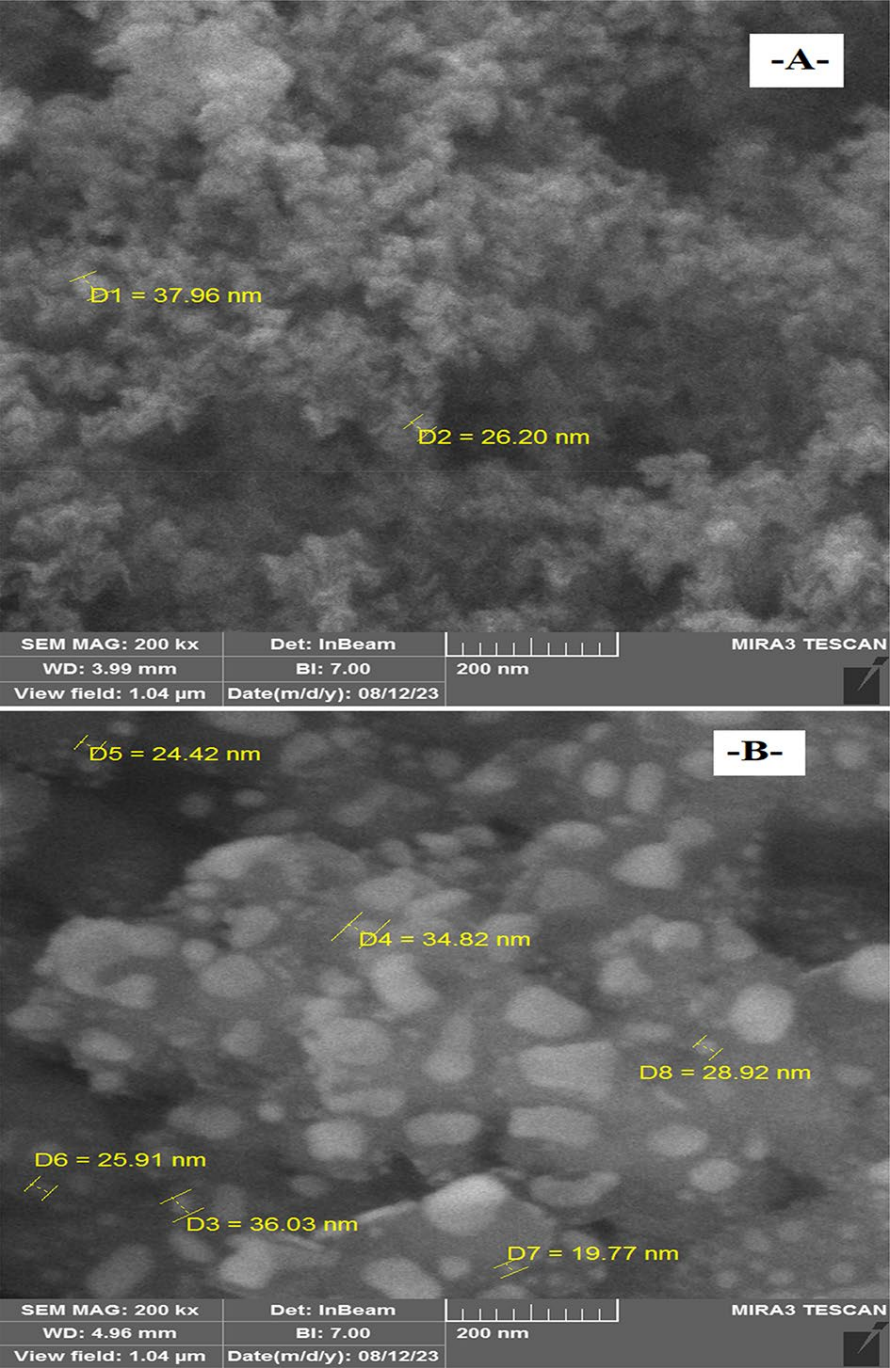
Pictures of FESEM. A: Silica nanoparticles; B: Magnesium oxide nanostructures.

This work employed FE-SEM to characterize nanomaterial morphology. Figure 3 shows high-magnification FE-SEM images of nanomaterials. These photos depict silica–magnesium oxide Nanoparticles (SNP + MgONP50/50) tubular morphology nicely and are similar to other conventional SEM images.

## Results and discussion

The Nanoparticles added to crude oil significantly reduced the viscosity. The viscosity was measured at different temperatures and at different concentrations at a constant shear rate to investigate its effect on the Degree of Viscosity Reduction (DVR).

**Figure 4:**
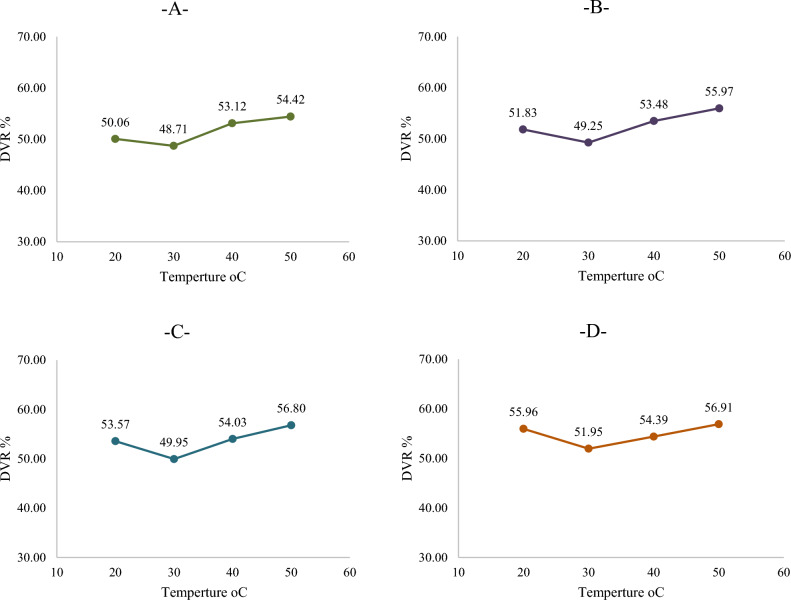
Effect of silica–magnesium oxide nanoparticles (SNP + MgONP50/50) on DVR percentages of Crude oil at different temperatures. (**A)** 0.1 wt% NP (SM1); (**B)** 0.2 wt% NP (SM2); (**C)** 0.3 wt% NP (SM3); (**D)** 0.4 wt% NP (SM4).

Figure 4 shows the effect of silica–magnesium oxide Nanoparticles (SNP + MgONP50/50) at different concentrations and temperatures in raising the DVR. The highest (DVR) (56.91%) was recorded at the highest concentration (0.4 wt%) (SM4) and at the highest temperature (50 °C). This is specified very well with^10^.

Several conditions and criteria were evaluated to analyse the change in the viscosity of crude oil samples resulting from the addition of Nanomaterials and to clarify the relationship that was found between the parameters shown in the associated figures to ascertain the effect of Nanomaterial additives on crude oil viscosity.

### Relationship between viscosity and nanoparticle concentration

Conducting tests for estimating the viscosity value of crude oil samples and other rheological properties before and after adding Nanomaterials is intended to understand the extent of the effect of changes in parameters on viscosity and rheological properties and the relationship between them directly and inversely.

**Figure 5:**
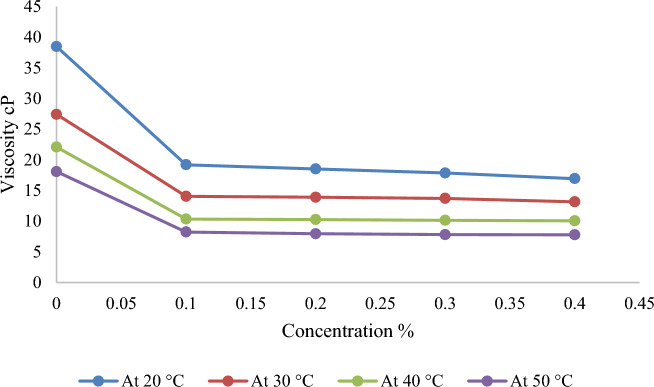
Variation of crude oil viscosity with different concentrations of (silica–magnesium oxide) nanoparticles (0.1, 0.2, 0.3, 0.4 wt%) (SNP + MgONP50/50) added at different temperatures.

Note that the additional Nanomaterials mixture concentration increases from 0.1, 0.2, 0.3, and 0.4 wt% for samples SM1, SM2, SM3, and SM4, respectively. Real-world tests show that all nanomaterials lower crude oil viscosity. With increasing concentration, the viscosity decreases further, as shown by the crude oil viscosity at 20 °C before adding 38.49 cP, which decreased to 19.22 cP for the sample SM1 and increased to 16.95 cP for the sample SM4. High temperatures contribute to reducing crude oil viscosity, but the effect of increasing the concentration of Nano-mixture is very clear, as the lowest viscosity was recorded by 7.8 cP at a temperature of 50 °C and a concentration of SM4 as shown in Fig. 5. Furthermore, increasing concentration lowers viscosity, however the abrupt drops after addition are somewhat different from those before addition. In the SM4 sample, increasing nanomaterial content by greater than 0.4% has not lowered the viscosity. This conclusion is specified very well with^11^.

### Relationship between viscosity and temperature

Given operating temperature is a crucial aspect in influencing the flow properties of crude oil, understanding how temperature affects crude oil viscosity is crucial. Consequently, the operating temperatures of 20, 30, 40, and 50 °C were selected based on the climate of Iraq.

**Figure 6:**
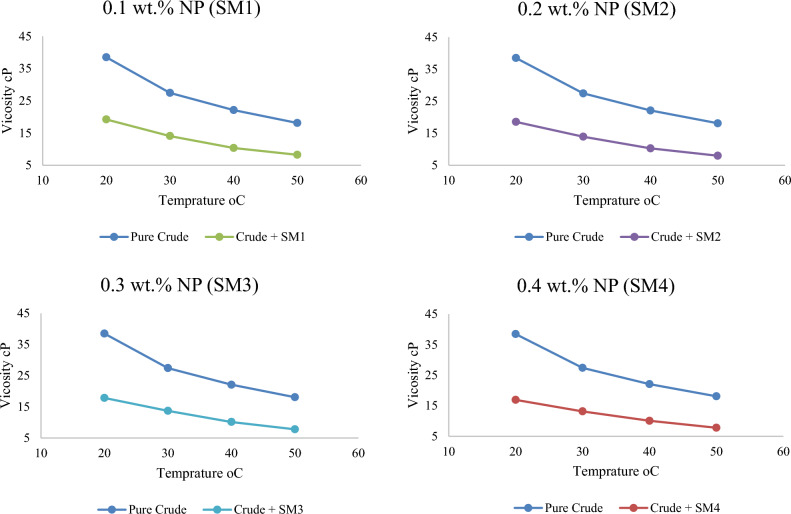
Variation of crude oil viscosity with different temperatures before and after adding silica–magnesium oxide Nanoparticles (SNP + MgONP50/50) in different concentrations.

**Figure 7:**
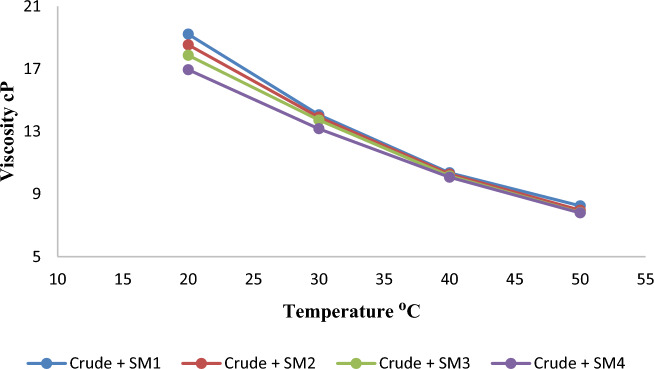
Variation of crude oil viscosity with different temperatures after adding silica–magnesium oxide Nanoparticles (SNP + MgONP50/50) in different concentrations.

As temperature rises, crude oil viscosity decreases. The average speed of molecules increases with temperature. As system temperature rises, average intermolecular forces decrease. Viscosity falls as stickiness and thickness decrease. The viscosity of the samples (before addition) decreased from 38.49 cP at 20 °C to 18.1 cP at 50 °C. Nevertheless, the impact of high temperature with the addition of Nanomaterials is higher and produces a bigger drop in the viscosity of crude oil by a substantial amount. This is shown by the fact that the viscosity reduces after adding the combination of Nanomaterials in the sample SM1, going from 19.22 cP at 20 °C to 8.25 cP at 50 °C. SM2 dropped 7.97 cP at 50 °C, SM3 7.82, and SM4 7.8. As demonstrated in Figs. 6 and 7, the regression decreases gradually as temperature increases, although it ranges between 20 and 30 °C. This result was also noticed by^12^.

### Relationship of viscosity with shear rate and shear stress

Shear stress and shear rate are the most important fluid rheological parameters for classifying crude oil as Newtonian or non-Newtonian. These metrics can be used to determine the rheology of pure crude oil and Nano-fluids.

**Figure 8:**
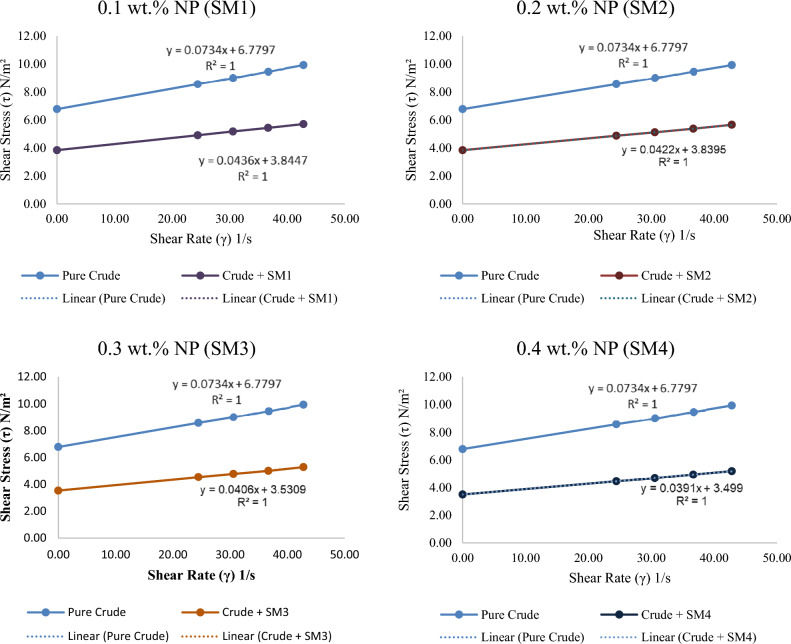
Relationship between shear stress with a variety of shear rates for crude oil before and after adding silica–magnesium oxide Nanoparticles (SNP + MgONP50/50) in different concentrations at 20 °C.

**Figure 9:**
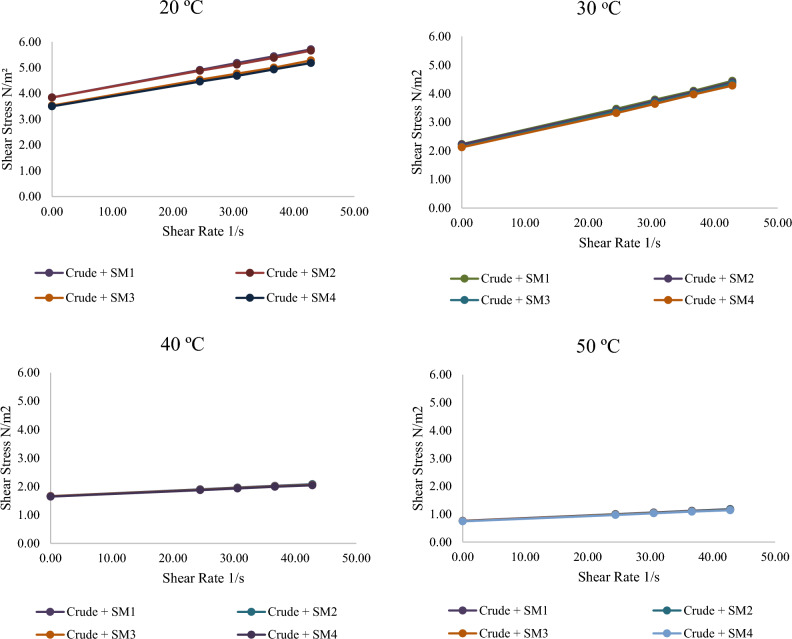
The relationship between shear stress with a variety of shear rates for crude oil after adding silica–magnesium oxide Nanoparticles (SNP + MgONP50/50).

**Figure 10:**
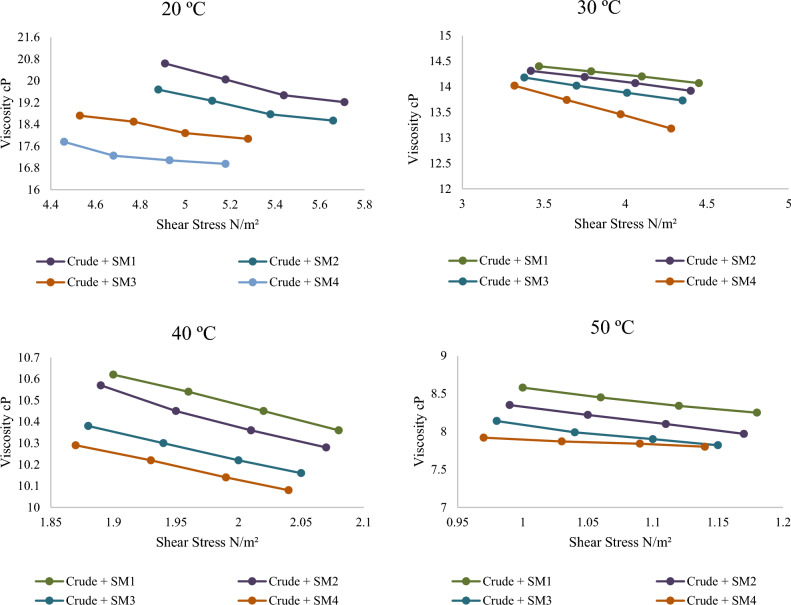
The relationship between viscosity of crude oil with a variety of shear rates after adding silica–magnesium oxide Nanoparticles (SNP + MgONP50/50) in different concentrations.

Illustrations show power-law plot rheology. Pure crude oil at different temperatures shows a linear increase in shear stress with shear rate. This concentration has the highest shear stress for any shear rate. At 0.4 wt% (SM4), silica–magnesium oxide Nanoparticles (SNP) concentration has the largest reduction and lowest shear stress. This is due to utilizing the highest concentration of 0.4 wt%. It is also noticed that the shear stress is minimised when the additive Nanoparticles mixture concentration is raised. This conclusion is specified very well with^13^. All the associated figures (Figs. 8, 9, 10) specify that pure crude oil and crude oil-based Nano-fluid behave Newtonian for all variables, with flow indices of unity (n = 1) for all operational temperatures, Nano-addition concentrations, and sonication periods. This means that the viscosity of Newtonian parent crude oil and crude oil-based Nano-fluids is temperature-dependent and does not alter with pipeline speed. In Figs. 8, 9 and 10, Nanoparticles decrease turbulence energy and lower shear stress, causing crude oil viscosity to fluctuate with increasing shear rates before and after adding them. Table 3 provides values of effective viscosity for crude oil before and after addition nanoparticles at different temperatures.

**Table 3:** Values of effective viscosity for crude oil before and after addition nanoparticles at different temperatures.

Sample	µ_eff_, cP (20 °C)	µ_eff_, cP (30 °C)	µ_eff_, cP (40 °C)	µ_eff_, cP (50 °C)
Pure Crude	84.2	70.7	28.7	28.2
SM1	49.7	44.9	17.4	13.3
SM2	48.3	44.2	17.3	13.2
SM3	46.2	42.7	16.8	13.2
SM4	44.6	41.7	16.7	13.1

It is noticeable from the obtained results that the liquid (crude oil) before adding Nano-mixture behaves according to the Bingham model, but after adding Nano-mixture with different concentrations, the yield stress value τ_y_ begins to decrease, as well as the value of the plastic viscosity µ^∞^. As a result, the effective viscosity µ_eff_ value decreases according to Eq. (6). Consequently, the SM4 sample has the greatest influence on the behaviour of crude oil, as shown in Table 3. As a result of the liquid's (crude oil) value decreasing and gradually approaching zero, the addition of Nanomaterials causes the liquid to behave more like a Newtonian system.

**Figure 11:**
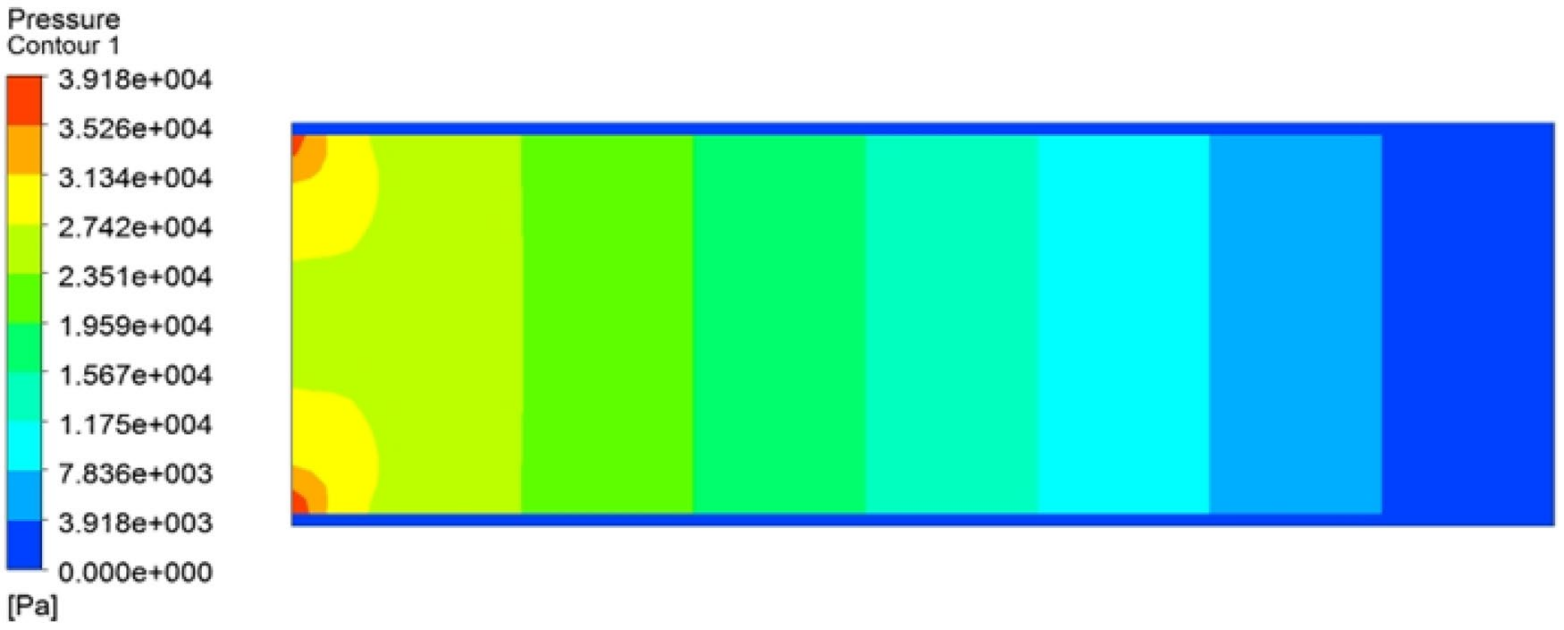
Contour of pressure distribution for crude oil (pure) at 20 °C.

It is also noted that the high temperature works to reduce the value of τ_y_ and µ^∞^, which helps in bringing the liquid behaviour closer to the Newtonian behaviour standard. Thus, the greatest effect on the behaviour of crude oil samples and their viscosity was recorded at the highest concentration of Nano-mixture added (SM4) and at the highest measured temperature (50 °C) (Figs. 8, 9, 10, 11). The lowest effective viscosity was recorded at 13.1 cP, and the lowest measured viscosity of the liquid under the same conditions was recorded at 7.8 cP. This is specified very well with^20^.

The coefficient of determination R^2^ is usually 1 in most cases, especially after adding Nanomaterials, indicating that this study's results can be used to predict future results or test hypotheses based on other relevant information. It also indicates how reproducible this model's outcomes are. R^2^ can also indicate the variables' correlation strength when 1 is attained.

Notably, we find that increasing shear stress causes a decrease in viscosity value, and the presence of Nanomaterials clearly increases the amount of this decrease in crude oil's viscosity value. This relationship between the obtained viscosity results and shear stress is noteworthy. In the same way, increasing shear rate decreases viscosity. Figure 13 shows that adding Nanoparticles has reduced crude oil viscosity.

### Flow system simulation

#### Pressure distribution

Simulation of crude oil flow characteristics by ANSYS Fluent v.16.1 software for pure crude oil, SM1, SM2, SM3, and SM4 samples were proposed to flow through a tube of stainless steel with an inner diameter of 15 cm and length of 50 cm. The boundary conditions are taken as follows: the inlet flow velocity is 1 m/s, the inlet fluid temperature is 20 °C, and the outlet pipe temperature is 55 °C.

The pressure distribution for the pure crude oil flow through the tube is depicted in Fig. 11. As the distance increases, the oil's velocity (or kinematic energy) decreases, which lowers pressure. This is the reason why pressure drops with increasing distance.

**Figure 12:**
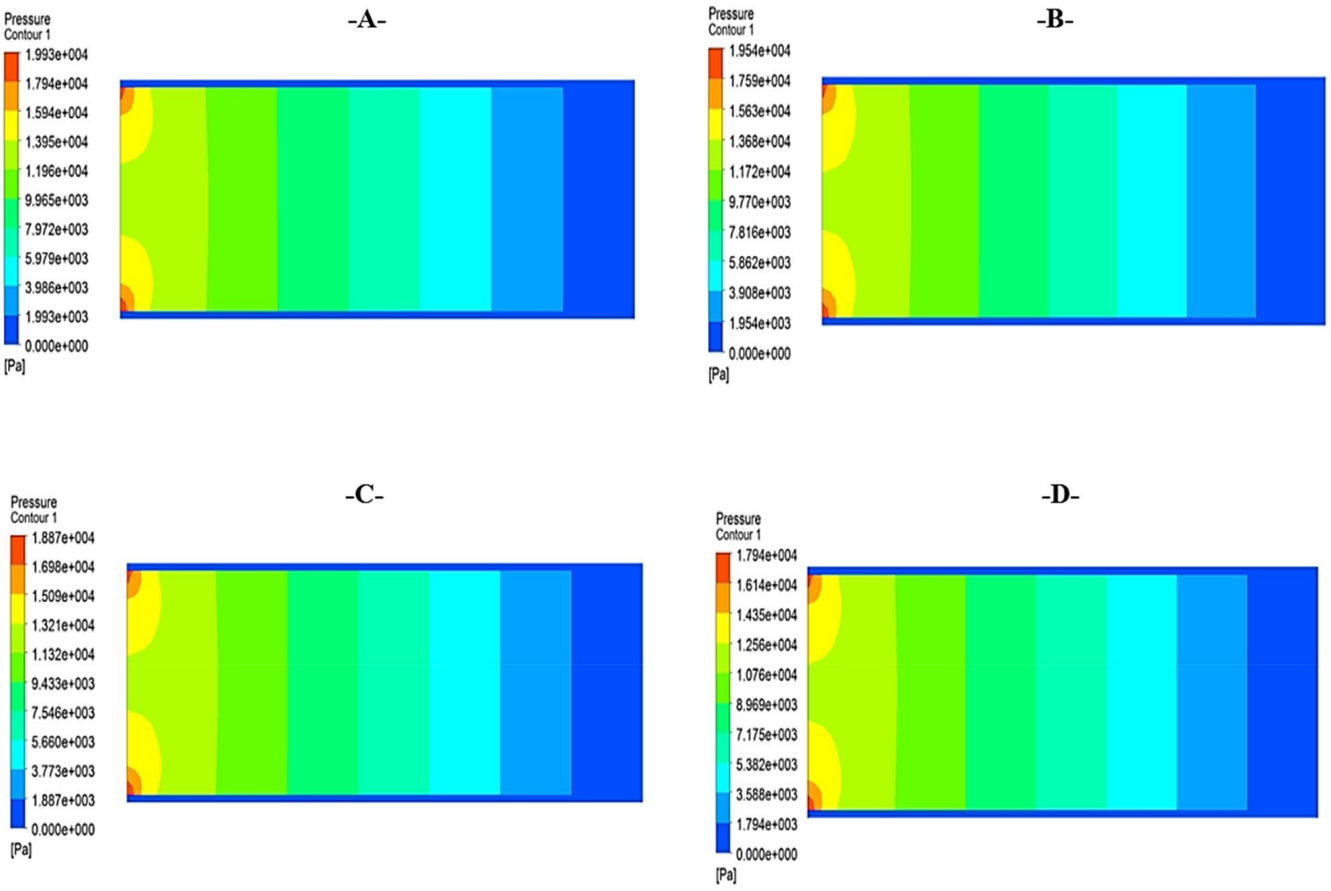
Contour of pressure distribution for silica–magnesium oxide Nanoparticles (SNP + MgONP50/50) by inlet flow velocity of 1 m/s at different temperatures.

**Figure 13:**
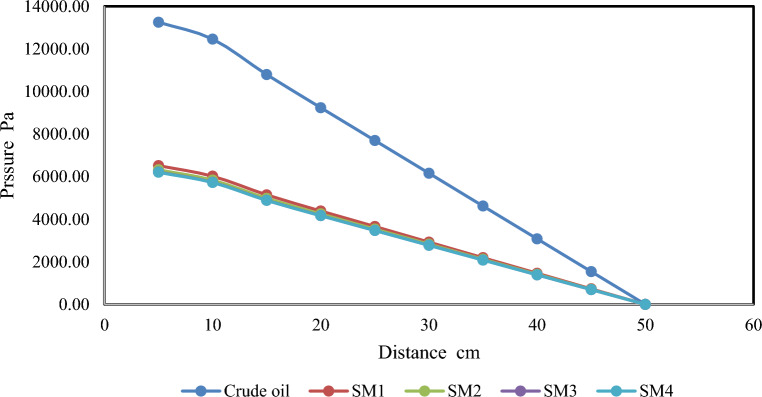
Variation of pressure distribution for crude oil, SM1, SM2, SM3, and SM4 at 20 °C.

Figure 12 presents the contours of pressure distribution for the flow of SM1, SM2, SM3, and SM4, respectively. It has been proved that a rise in the Nanoparticle volume fractions results in a lower pressure distribution throughout the system. This is due to the fact that the incorporation of Nanoparticles causes a reduction in viscosity, which, in turn, leads to a rise in the Reynolds number. This, in turn, brings about a reduction in the friction factor, which ultimately brings about a reduction in the pressure drop. The pressure distribution has been reduced by adding the Nanoparticles for sample (SM1), and there is no noticeable increase in the pressure reduction for SM2, SM3, and SM4, which can be detected in Fig. 13. This is specified very well according to^20^.

### Drag reduction

**Figure 14:**
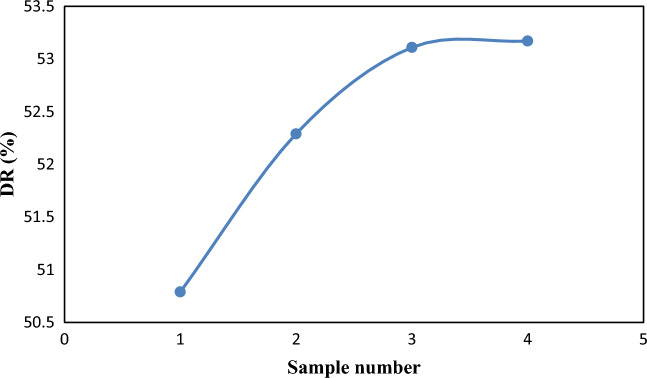
Variation of drag reduction for the four samples (SM1, SM2, SM3, and SM4) at 20 °C.

The drag reduction variation for the four samples (SM1, SM2, SM3, and SM4) at 20 °C and an input flow velocity of 1 m/s is shown in Fig. 14. It is evident that a higher volume proportion of nanoparticles would result in a greater reduction of drag. SM4 has the greatest drag decrease, which is 53.17%. This phenomenon was also seen by^16^.

### Flow increase (FI)

**Figure 15:**
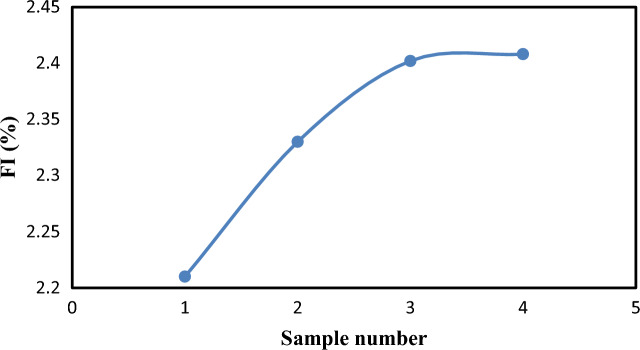
Variation of flow increase for the four samples (SM1, SM2, SM3, and SM4) at 20 °C.

An increase in flow is one of the extra advantages of including the Nanoparticles, as seen in Fig. 15. The sample SM4 was found to have the maximum value of flow increase, which is 2.408%. This is specified very well with^18^.

### Friction factor

**Figure 16:**
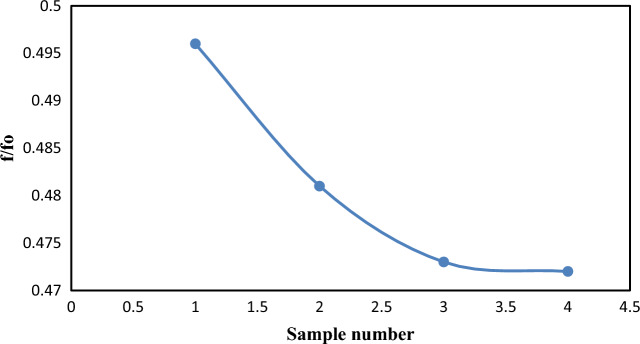
Variation of friction factor ratio for the four samples (SM1, SM2, SM3, and SM4) at 20 °C.

Figure 16 shows the value of the friction factor for the addition of Nanoparticles (f) to the friction factor for pure crude oil (fo). The introduction of Nanoparticles into crude oil has been shown to decrease the oil's viscosity, which in turn has decreased the friction factor ratio. This is specified very well according to^20^.

## Conclusions

By actually absorbing the nanoparticles into the rock surfaces, the usage of nanoparticles and their characteristics—such as wettability, surface tension reduction, and viscosity reduction between the oil and water surfaces—can be very helpful in enhancing the recovery efficiency. The size of the particles and their capacity to alter and control liquid phase behavior are two characteristics of Nanoparticles that make them intriguing for enhancing over-harvesting procedures.

According to the findings of the current study, the addition of Nanomaterial has resulted in a significant reduction in viscosity, and the lowest viscosity values were recorded (7.8 cP) at a concentration of 0.4 wt% (SM4) of Nano-mixture. The degree of viscosity reduction was detected as a function of the kind of Nanomaterial, their concentration, temperature, and sonication time, where the highest degree of viscosity reduction (DVR) was recorded (56.91%) at the highest concentration (0.4 wt%) and at the highest temperature (50 °C). Furthermore, it was noted that the liquid (crude oil) behaves as predicted by the Bingham model prior to the addition of Nano-mixture. However, yield stress value τy, plastic viscosity µ∞, and effective viscosity µeff all start to decrease following the addition of Nano-mixture at varying concentrations. This indicates that the addition of nanomaterials to crude oil causes the liquid to behave more like a Newtonian system in addition to raising the temperature because of the slow approach of zero for τy and its subsequent decrease in value.

The numerical results demonstrate that pressure drops with increasing distance, and that the pressure distribution decreased as the nanoparticle volume percentage increased. Furthermore, adding nanoparticles to sample (SM1) resulted in a decrease in the pressure distribution; however, pressure reductions for samples (SM2), (SM3), and (SM4) did not show any discernible improvement. Moreover, an increase in the volume percentage of nanoparticles has resulted in a greater reduction in drag. The maximum drag reduction of 53.17% was achieved for SM4. The maximum value of flow increase is 2.408%, which was also obtained for SM4. Lastly, the viscosity of crude oil decreases as a result of the introduction of Nanoparticles, lowering the friction factor ratio.

### Supplementary Information

Supplementary Information.

## Data Availability

All data generated or analysed during this study are included in this published article [and its supplementary information files].
