# Supplementary material for: An experimental investigation and flow-system simulation about the influencing of silica–magnesium oxide nano-mixture on enhancing the rheological properties of Iraqi crude oil
Source: Sci Rep. 2024 Mar 14;14:6148. doi: 10.1038/s41598-024-56722-x (PMC10937623; doi:10.1038/s41598-024-56722-x)
Supplement: Supplementary file 1 — Supplementary Information. [file 41598_2024_56722_MOESM1_ESM.pdf]

Data Report

Sample Information:

Sample Name: {pure 20C}

Tester Name: Salem Alhamd **Torque Coefficient:** 0.0937300026 **Result Notes:**

Run Time: 01/12/2022

Instr. S/N: 0

Torque Range: LV

Test Method:

Test Name {pure 20C}

Saved On: 01/12/2022 12:17:00 AM **Math Model:** None **Instruction**

Spindle: DIN-87 **Avg. Steps:** False

# of Steps: 4 **Avg. Test:** False

# of Loops: 0

Use Path: False **Result Path:**

Step#	Spd (rpm)	Inc. Spd	Use Tmp	Tmp (C°)	Inc. Tmp	Data Type	Data intr (sec)	Avr Dur (sec)	Point@ End	End Type	End Value	End Tol.	Density (g/cm3)	QCtype	QCL		QCH		In Test Avr.
1	20	True	False	20	False	Single	0	0	False	Time	60	0	0.00	None	0.00	%	0.00	%	False
2	25	False	False	20	False	Single	0	0	False	Time	60	0	0.00	None	0.00	%	0.00	%	False
3	30	False	False	20	False	Single	0	0	False	Time	60	0	0.00	None	0.00	%	0.00	%	False
4	35	False	False	20	False	Single	0	0	False	Time	60	0	0.00	None	0.00	%	0.00	%	False

Test Data:

Data Point	Step Point	Speed RPM	Torque (%)	Temperature °C	Time (Sec)	Viscosity cP	Shear Stress N/m²	Shear Rate 1/s
1	1	20.00	48.6	20.0	00:01:31.0	39.12	8.58	24.46
2	2	25.00	60.0	20.0	00:02:24.1	38.78	9.01	30.58
3	3	30.00	72.1	20.0	00:03:41.0	38.66	9.47	36.69
4	4	35.00	84.4	20.0	00:04:45.0	38.49	9.93	42.82

Data Report

Sample Information:

Sample Name: {SM1 20C}

Tester Name: Salem Alhamd **Torque Coefficient:** 0.0937300026 **Result Notes:**
Run Time: 01/12/2022
Instr. S/N: 0
Torque Range: LV

Test Method:

Test Name {SM1 20C}

Saved On: 01/12/2022 1:42:00 PM **Math Model:** None **Instruction** **Instruction**
Spindle: DIN-87 **Avg. Steps:** False
# of Steps: 4 **Avg. Test:** False
# of Loops: 0
Use Path: False **Result Path:**

Step#	Spd (rpm)	Inc. Spd	Use Tmp	Tmp (C°)	Inc. Tmp	Data Type	Data intr (sec)	Avr Dur (sec)	Point@ End	End Type	End Value	End Tol.	Density (g/cm3)	QCtype	QCL		QCH		In Test Avr.
1	20	True	False	20	False	Single	0	0	False	Time	60	0	0.00	None	0.00	%	0.00	%	False
2	25	False	False	20	False	Single	0	0	False	Time	60	0	0.00	None	0.00	%	0.00	%	False
3	30	False	False	20	False	Single	0	0	False	Time	60	0	0.00	None	0.00	%	0.00	%	False
4	35	False	False	20	False	Single	0	0	False	Time	60	0	0.00	None	0.00	%	0.00	%	False

Test Data:

Data Point	Step Point	Speed RPM	Torque (%)	Temperature °C	Time (Sec)	Viscosity cP	Shear Stress N/m²	Shear Rate 1/s
1	1	20.00	44.0	20.0	00:01:22.0	20.64	4.91	24.46
2	2	25.00	52.3	20.0	00:02:34.0	20.05	5.18	30.58
3	3	30.00	61.0	20.0	00:03:27.0	19.26	5.44	36.69
4	4	35.00	67.7	20.0	00:04:39.0	19.22	5.71	42.82

Rheocalc T V1.0.1
Brookfield Engineering Labs INC.

Data Report

Sample Information: **Sample Name:** {SM2 20C}

Tester Name: Salem Alhamd **Torque Coefficient:** 0.0937300026 **Result Notes:**
Run Time: 01/12/2022
Instr. S/N: 0
Torque Range: LV

Test Method: **Test Name** {SM2 20C}

Saved On: 01/12/2022 2:30:00 PM **Math Model:** None **Instruction** **Instruction**
Spindle: DIN-87 **Avg. Steps:** False
# of Steps: 4 **Avg. Test:** False
# of Loops: 0
Use Path: False **Result Path:**

Step#	Spd (rpm)	Inc. Spd	Use Tmp	Tmp (C°)	Inc. Tmp	Data Type	Data intr (sec)	Avr Dur (sec)	Point@ End	End Type	End Value	End Tol.	Density (g/cm3)	QCtype	QCL		QCH		In Test Avr.
1	20	True	False	20	False	Single	0	0	False	Time	60	0	0.00	None	0.00	%	0.00	%	False
2	25	False	False	20	False	Single	0	0	False	Time	60	0	0.00	None	0.00	%	0.00	%	False
3	30	False	False	20	False	Single	0	0	False	Time	60	0	0.00	None	0.00	%	0.00	%	False
4	35	False	False	20	False	Single	0	0	False	Time	60	0	0.00	None	0.00	%	0.00	%	False

Test Data:

Data Point	Step Point	Speed RPM	Torque (%)	Temperature °C	Time (Sec)	Viscosity cP	Shear Stress N/m²	Shear Rate 1/s
1	1	20.00	44.2	20.0	00:01:21.0	19.68	4.88	24.46
2	2	25.00	51.4	20.0	00:02:31.0	19.27	5.12	30.58
3	3	30.00	60.8	20.0	00:03:42.0	18.77	5.38	36.69
4	4	35.00	67.3	20.0	00:04:56.0	18.54	5.66	42.82

Rheocalc T V1.0.1

Brookfield Engineering Labs INC.

Data Report

Sample Information: **Sample Name:** {SM3 20C}

Tester Name: Salem Alhamd **Torque Coefficient:** 0.0937300026 **Result Notes:**

Run Time: 01/12/2022

Instr. S/N: 0

Torque Range: LV

Test Method: **Test Name** {SM3 20C}

Saved On: 01/12/2022 2:47:00 PM **Math Model:** None **Instruction** **Instruction**

Spindle: DIN-87 **Avg. Steps:** False

# of Steps: 4 **Avg. Test:** False

# of Loops: 0

Use Path: False **Result Path:**

Step#	Spd (rpm)	Inc. Spd	Use Tmp	Tmp (C°)	Inc. Tmp	Data Type	Data intr (sec)	Avr Dur (sec)	Point@ End	End Type	End Value	End Tol.	Density (g/cm3)	QCtype	QCL		QCH		In Test Avr.
1	20	True	False	20	False	Single	0	0	False	Time	60	0	0.00	None	0.00	%	0.00	%	False
2	25	False	False	20	False	Single	0	0	False	Time	60	0	0.00	None	0.00	%	0.00	%	False
3	30	False	False	20	False	Single	0	0	False	Time	60	0	0.00	None	0.00	%	0.00	%	False
4	35	False	False	20	False	Single	0	0	False	Time	60	0	0.00	None	0.00	%	0.00	%	False

Test Data:

Data Point	Step Point	Speed RPM	Torque (%)	Temperature °C	Time (Sec)	Viscosity cP	Shear Stress N/m²	Shear Rate 1/s
1	1	20.00	43.4	20.0	00:01:23.0	18.72	4.53	24.46
2	2	25.00	50.2	20.0	00:02:31.0	18.50	4.77	30.58
3	3	30.00	58.6	20.0	00:03:37.0	18.08	5.00	36.69
4	4	35.00	66.2	20.0	00:04:41.0	17.87	5.28	42.82

Rheocalc T V1.0.1

Brookfield Engineering Labs INC.

Data Report

Sample Information: **Sample Name:** {SM4 20C}

Tester Name: Salem Alhamd **Torque Coefficient:** 0.0937300026 **Result Notes:**

Run Time: 01/12/2022

Instr. S/N: 0

Torque Range: LV

Test Method: **Test Name** {SM4 20C}

Saved On: 01/12/2022 3:00:00 PM **Math Model:** None **Instruction** **Instruction**

Spindle: DIN-87 **Avg. Steps:** False

# of Steps: 4 **Avg. Test:** False

# of Loops: 0

Use Path: False **Result Path:**

Step#	Spd (rpm)	Inc. Spd	Use Tmp	Tmp (C°)	Inc. Tmp	Data Type	Data intr (sec)	Avr Dur (sec)	Point@ End	End Type	End Value	End Tol.	Density (g/cm3)	QCtype	QCL	QCH	In Test Avr.
1	20	True	False	20	False	Single	0	0	False	Time	60	0	0.00	None	0.00 %	0.00 %	False
2	25	False	False	20	False	Single	0	0	False	Time	60	0	0.00	None	0.00 %	0.00 %	False
3	30	False	False	20	False	Single	0	0	False	Time	60	0	0.00	None	0.00 %	0.00 %	False
4	35	False	False	20	False	Single	0	0	False	Time	60	0	0.00	None	0.00 %	0.00 %	False

Test Data:

Data Point	Step Point	Speed RPM	Torque (%)	Temperature °C	Time (Sec)	Viscosity cP	Shear Stress N/m²	Shear Rate 1/s
1	1	20.00	42.4	20.0	00:01:21.0	17.76	4.46	24.46
2	2	25.00	49.9	20.0	00:02:26.0	17.25	4.68	30.58
3	3	30.00	57.6	20.0	00:03:38.0	17.08	4.93	36.69
4	4	35.00	65.3	20.0	00:04:46.0	16.95	5.18	42.82

Data Report

Sample Information: **Sample Name:** {pure 30C}

Tester Name: Salem Alhamd **Torque Coefficient:** 0.0937300026 **Result Notes:**
Run Time: 02/12/2022
Instr. S/N: 0
Torque Range: LV

Test Method: **Test Name** {pure 30C}

Saved On: 02/12/2022 10:14:00 AM **Math Model:** None **Instruction** **Instruction**
Spindle: DIN-87 **Avg. Steps:** False
# of Steps: 4 **Avg. Test:** False
# of Loops: 0
Use Path: False **Result Path:**

Step#	Spd (rpm)	Inc. Spd	Use Tmp	Tmp (C°)	Inc. Tmp	Data Type	Data intr (sec)	Avr Dur (sec)	Point@ End	End Type	End Value	End Tol.	Density (g/cm3)	QCtype	QCL		QCH		In Test Avr.
1	20	True	False	30	False	Single	0	0	False	Time	60	0	0.00	None	0.00	%	0.00	%	False
2	25	False	False	30	False	Single	0	0	False	Time	60	0	0.00	None	0.00	%	0.00	%	False
3	30	False	False	30	False	Single	0	0	False	Time	60	0	0.00	None	0.00	%	0.00	%	False
4	35	False	False	30	False	Single	0	0	False	Time	60	0	0.00	None	0.00	%	0.00	%	False

Test Data:

Data Point	Step Point	Speed RPM	Torque (%)	Temperature °C	Time (Sec)	Viscosity cP	Shear Stress N/m²	Shear Rate 1/s
1	1	20.00	44.3	30.0	00:01:30.0	29.68	4.77	24.46
2	2	25.00	51.6	30.0	00:02:23.0	28.45	5.03	30.58
3	3	30.00	59.5	30.0	00:03:21.0	27.96	5.35	36.69
4	4	35.00	67.5	30.0	00:04:43.0	27.43	5.66	42.82

Data Report

Sample Information: **Sample Name:** {SM1 30C}

Tester Name: Salem Alhamd **Torque Coefficient:** 0.0937300026 **Result Notes:**
Run Time: 02/12/2022
Instr. S/N: 0
Torque Range: LV

Test Method: **Test Name** {SM1 30C}

Saved On: 02/12/2022 11:37:00 AM **Math Model:** None **Instruction** **Instruction**
Spindle: DIN-87 **Avg. Steps:** False
# of Steps: 4 **Avg. Test:** False
# of Loops: 0
Use Path: False **Result Path:**

Step#	Spd (rpm)	Inc. Spd	Use Tmp	Tmp (C°)	Inc. Tmp	Data Type	Data intr (sec)	Avr Dur (sec)	Point@ End	End Type	End Value	End Tol.	Density (g/cm3)	QCtype	QCL		QCH		In Test Avr.
1	20	True	False	30	False	Single	0	0	False	Time	60	0	0.00	None	0.00	%	0.00	%	False
2	25	False	False	30	False	Single	0	0	False	Time	60	0	0.00	None	0.00	%	0.00	%	False
3	30	False	False	30	False	Single	0	0	False	Time	60	0	0.00	None	0.00	%	0.00	%	False
4	35	False	False	30	False	Single	0	0	False	Time	60	0	0.00	None	0.00	%	0.00	%	False

Test Data:

Data Point	Step Point	Speed RPM	Torque (%)	Temperature °C	Time (Sec)	Viscosity cP	Shear Stress N/m²	Shear Rate 1/s
1	1	20.00	39.2	30.0	00:01:41.0	14.40	3.47	24.46
2	2	25.00	47.3	30.0	00:02:37.0	14.30	3.79	30.58
3	3	30.00	56.0	30.0	00:03:28.0	14.20	4.08	36.69
4	4	35.00	61.7	30.0	00:04:30.0	14.07	4.33	42.82

Data Report

Sample Information:

Sample Name: {SM2 30C}

Tester Name: Salem Alhamd **Torque Coefficient:** 0.0937300026 **Result Notes:**

Run Time: 02/12/2022

Instr. S/N: 0

Torque Range: LV

Test Method:

Test Name {SM2 30C}

Saved On: 02/12/2022 11:50:00 AM **Math Model:** None **Instruction** **Instruction**

Spindle: DIN-87 **Avg. Steps:** False

# of Steps: 4 **Avg. Test:** False

# of Loops: 0

Use Path: False **Result Path:**

Step#	Spd (rpm)	Inc. Spd	Use Tmp	Tmp (C°)	Inc. Tmp	Data Type	Data intr (sec)	Avr Dur (sec)	Point@ End	End Type	End Value	End Tol.	Density (g/cm3)	QCtype	QCL		QCH		In Test Avr.
1	20	True	False	30	False	Single	0	0	False	Time	60	0	0.00	None	0.00	%	0.00	%	False
2	25	False	False	30	False	Single	0	0	False	Time	60	0	0.00	None	0.00	%	0.00	%	False
3	30	False	False	30	False	Single	0	0	False	Time	60	0	0.00	None	0.00	%	0.00	%	False
4	35	False	False	30	False	Single	0	0	False	Time	60	0	0.00	None	0.00	%	0.00	%	False

Test Data:

Data Point	Step Point	Speed RPM	Torque (%)	Temperature °C	Time (Sec)	Viscosity cP	Shear Stress N/m²	Shear Rate 1/s
1	1	20.00	39.0	30.0	00:01:23.0	14.31	3.42	24.46
2	2	25.00	46.5	30.0	00:02:45.0	14.19	3.71	30.58
3	3	30.00	55.2	30.0	00:03:26.0	14.07	3.99	36.69
4	4	35.00	61.2	30.0	00:04:48.0	13.92	4.23	42.82

Data Report

Sample Information: **Sample Name:** {SM3 30C}

Tester Name: Salem Alhamd **Torque Coefficient:** 0.0937300026 **Result Notes:**
Run Time: 02/12/2022
Instr. S/N: 0
Torque Range: LV

Test Method: **Test Name** {SM3 30C}

Saved On: 02/12/2022 12:05:00 AM **Math Model:** None **Instruction** **Instruction**
Spindle: DIN-87 **Avg. Steps:** False
# of Steps: 4 **Avg. Test:** False
# of Loops: 0
Use Path: False **Result Path:**

Step#	Spd (rpm)	Inc. Spd	Use Tmp	Tmp (C°)	Inc. Tmp	Data Type	Data intr (sec)	Avr Dur (sec)	Point@ End	End Type	End Value	End Tol.	Density (g/cm3)	QCtype	QCL		QCH		In Test Avr.
1	20	True	False	30	False	Single	0	0	False	Time	60	0	0.00	None	0.00	%	0.00	%	False
2	25	False	False	30	False	Single	0	0	False	Time	60	0	0.00	None	0.00	%	0.00	%	False
3	30	False	False	30	False	Single	0	0	False	Time	60	0	0.00	None	0.00	%	0.00	%	False
4	35	False	False	30	False	Single	0	0	False	Time	60	0	0.00	None	0.00	%	0.00	%	False

Test Data:

Data Point	Step Point	Speed RPM	Torque (%)	Temperature °C	Time (Sec)	Viscosity cP	Shear Stress N/m²	Shear Rate 1/s
1	1	20.00	38.8	30.0	00:01:15.0	14.18	3.37	24.46
2	2	25.00	45.7	30.0	00:02:26.0	14.02	3.62	30.58
3	3	30.00	54.4	30.0	00:03:37.0	13.88	3.90	36.69
4	4	35.00	60.9	30.0	00:04:28.0	13.73	4.14	42.82

Data Report

Sample Information:

Sample Name: {SM4 30C}

Tester Name: Salem Alhamd **Torque Coefficient:** 0.0937300026 **Result Notes:**

Run Time: 02/12/2022

Instr. S/N: 0

Torque Range: LV

Test Method:

Test Name {SM4 30C}

Saved On: 02/12/2022 12:22:00 AM **Math Model:** None **Instruction** **Instruction**

Spindle: DIN-87 **Avg. Steps:** False

# of Steps: 4 **Avg. Test:** False

# of Loops: 0

Use Path: False **Result Path:**

Step#	Spd (rpm)	Inc. Spd	Use Tmp	Tmp (C°)	Inc. Tmp	Data Type	Data intr (sec)	Avr Dur (sec)	Point@ End	End Type	End Value	End Tol.	Density (g/cm3)	QCtype	QCL		QCH		In Test Avr.
1	20	True	False	30	False	Single	0	0	False	Time	60	0	0.00	None	0.00	%	0.00	%	False
2	25	False	False	30	False	Single	0	0	False	Time	60	0	0.00	None	0.00	%	0.00	%	False
3	30	False	False	30	False	Single	0	0	False	Time	60	0	0.00	None	0.00	%	0.00	%	False
4	35	False	False	30	False	Single	0	0	False	Time	60	0	0.00	None	0.00	%	0.00	%	False

Test Data:

Data Point	Step Point	Speed RPM	Torque (%)	Temperature °C	Time (Sec)	Viscosity cP	Shear Stress N/m²	Shear Rate 1/s
1	1	20.00	38.2	30.0	00:01:45.0	14.02	3.27	24.46
2	2	25.00	45.5	30.0	00:02:34.0	13.74	3.51	30.58
3	3	30.00	53.2	30.0	00:03:27.0	13.46	3.80	36.69
4	4	35.00	58.7	30.0	00:04:42.0	13.18	4.04	42.82

Data Report

Sample Information:

Sample Name: {pure 40C}

Tester Name: Salem Alhamd **Torque Coefficient:** 0.0937300026 **Result Notes:**

Run Time: 03/12/2022

Instr. S/N: 0

Torque Range: LV

Test Method:

Test Name {pure 40C}

Saved On: 03/12/2022 10:31:00 AM **Math Model:** None **Instruction** **Instruction**

Spindle: DIN-87 **Avg. Steps:** False

# of Steps: 4 **Avg. Test:** False

# of Loops: 0

Use Path: False **Result Path:**

Step#	Spd (rpm)	Inc. Spd	Use Tmp	Tmp (C°)	Inc. Tmp	Data Type	Data intr (sec)	Avr Dur (sec)	Point@ End	End Type	End Value	End Tol.	Density (g/cm3)	QCtype	QCL	QCH	In Test Avr.
1	20	False	False	40	False	Single	0	0	False	Time	90	0	0.00	None	0.00 %	0.00 %	False
2	25	False	False	40	False	Single	0	0	False	Time	90	0	0.00	None	0.00 %	0.00 %	False
3	30	False	False	40	False	Single	0	0	False	Time	90	0	0.00	None	0.00 %	0.00 %	False
4	35	False	False	40	False	Single	0	0	False	Time	90	0	0.00	None	0.00 %	0.00 %	False

Test Data:

Data Point	Step Point	Speed RPM	Torque (%)	Temperature °C	Time (Sec)	Viscosity cP	Shear Stress N/m²	Shear Rate 1/s
1	1	20.00	40.6	40.0	00:01:30.1	23.58	3.56	24.46
2	2	25.00	47.0	40.0	00:03:00.1	22.80	3.64	30.58
3	3	30.00	55.1	40.0	00:04:30.1	22.26	3.73	36.69
4	4	35.00	63.4	40.0	00:06:00.1	22.10	3.82	42.82

Data Report

Sample Information:

Sample Name: {SM1 40C}

Tester Name: Salem Alhamd **Torque Coefficient:** 0.0937300026 **Result Notes:**

Run Time: 03/12/2022

Instr. S/N: 0

Torque Range: LV

Test Method:

Test Name {SM1 40C}

Saved On: 03/12/2022 12:10:00 AM **Math Model:** None **Instruction** **Instruction**

Spindle: DIN-87 **Avg. Steps:** False

# of Steps: 4 **Avg. Test:** False

# of Loops: 0

Use Path: False **Result Path:**

Step#	Spd (rpm)	Inc. Spd	Use Tmp	Tmp (C°)	Inc. Tmp	Data Type	Data intr (sec)	Avr Dur (sec)	Point@ End	End Type	End Value	End Tol.	Density (g/cm3)	QCtype	QCL	QCH	In Test Avr.
1	20	False	False	40	False	Single	0	0	False	Time	90	0	0.00	None	0.00 %	0.00 %	False
2	25	False	False	40	False	Single	0	0	False	Time	90	0	0.00	None	0.00 %	0.00 %	False
3	30	False	False	40	False	Single	0	0	False	Time	90	0	0.00	None	0.00 %	0.00 %	False
4	35	False	False	40	False	Single	0	0	False	Time	90	0	0.00	None	0.00 %	0.00 %	False

Test Data:

Data Point	Step Point	Speed RPM	Torque (%)	Temperature °C	Time (Sec)	Viscosity cP	Shear Stress N/m²	Shear Rate 1/s
1	1	20.00	37.3	40.0	00:00:39.2	10.62	1.90	24.46
2	2	25.00	45.6	40.0	00:02:17.4	10.54	1.96	30.58
3	3	30.00	52.0	40.0	00:03:28.8	10.45	2.02	36.69
4	4	35.00	59.5	40.0	00:05:15.5	10.36	2.08	42.82

Rheocalc T V1.0.1

Brookfield Engineering Labs INC.

Data Report

Sample Information: **Sample Name:** {SM2 40C}

Tester Name: Salem Alhamd **Torque Coefficient:** 0.0937300026 **Result Notes:**

Run Time: 03/12/2022

Instr. S/N: 0

Torque Range: LV

Test Method: **Test Name** {SM2 40C}

Saved On: 03/12/2022 12:25:00 AM **Math Model:** None **Instruction** **Instruction**

Spindle: DIN-87 **Avg. Steps:** False

# of Steps: 4 **Avg. Test:** False

# of Loops: 0

Use Path: False **Result Path:**

Step#	Spd (rpm)	Inc. Spd	Use Tmp	Tmp (C°)	Inc. Tmp	Data Type	Data intr (sec)	Avr Dur (sec)	Point@ End	End Type	End Value	End Tol.	Density (g/cm3)	QCtype	QCL		QCH		In Test Avr.
1	20	False	False	40	False	Single	0	0	False	Time	90	0	0.00	None	0.00	%	0.00	%	False
2	25	False	False	40	False	Single	0	0	False	Time	90	0	0.00	None	0.00	%	0.00	%	False
3	30	False	False	40	False	Single	0	0	False	Time	90	0	0.00	None	0.00	%	0.00	%	False
4	35	False	False	40	False	Single	0	0	False	Time	90	0	0.00	None	0.00	%	0.00	%	False

Test Data:

Data Point	Step Point	Speed RPM	Torque (%)	Temperature °C	Time (Sec)	Viscosity cP	Shear Stress N/m²	Shear Rate 1/s
1	1	20.00	36.3	40.0	00:03:03.0	10.57	1.89	24.46
2	2	44.60	44.6	40.0	00:05:33.9	10.45	1.95	30.58
3	3	53.20	51.2	40.0	00:09:45.2	10.36	2.01	36.69
4	4	62.10	58.1	40.0	00:12:26.7	10.28	2.07	42.82

Data Report

Sample Information:

Sample Name: {SM3 40C}

Tester Name: Salem Alhamd **Torque Coefficient:** 0.0937300026 **Result Notes:**

Run Time: 03/12/2022

Instr. S/N: 0

Torque Range: LV

Test Method:

Test Name {SM3 40C}

Saved On: 03/12/2022 12:45:00 AM **Math Model:** None **Instruction** **Instruction**

Spindle: DIN-87 **Avg. Steps:** False

# of Steps: 4 **Avg. Test:** False

# of Loops: 0

Use Path: False **Result Path:**

Step#	Spd (rpm)	Inc. Spd	Use Tmp	Tmp (C°)	Inc. Tmp	Data Type	Data intr (sec)	Avr Dur (sec)	Point@ End	End Type	End Value	End Tol.	Density (g/cm3)	QCtype	QCL		QCH		In Test Avr.
1	20	False	False	40	False	Single	0	0	False	Time	90	0	0.00	None	0.00	%	0.00	%	False
2	25	False	False	40	False	Single	0	0	False	Time	90	0	0.00	None	0.00	%	0.00	%	False
3	30	False	False	40	False	Single	0	0	False	Time	90	0	0.00	None	0.00	%	0.00	%	False
4	35	False	False	40	False	Single	0	0	False	Time	90	0	0.00	None	0.00	%	0.00	%	False

Test Data:

Data Point	Step Point	Speed RPM	Torque (%)	Temperature °C	Time (Sec)	Viscosity cP	Shear Stress N/m²	Shear Rate 1/s
1	1	20.00	35.3	40.0	00:02:25.1	10.38	1.88	24.46
2	2	25.00	43.2	40.0	00:04:07.5	10.30	1.94	30.58
3	3	30.00	50.6	40.1	00:06:23.3	10.22	2.00	36.69
4	4	35.00	57.0	40.1	00:08:17.0	10.16	2.05	42.82

Rheocalc T V1.0.1
Brookfield Engineering Labs INC.

Data Report

Sample Information: **Sample Name:** {SM4 40C}

Tester Name: Salem Alhamd **Torque Coefficient:** 0.0937300026 **Result Notes:**
Run Time: 03/12/2022
Instr. S/N: 0
Torque Range: LV

Test Method: **Test Name** {SM4 40C}

Saved On: 03/12/2022 1:03:00 PM **Math Model:** None **Instruction** **Instruction**
Spindle: DIN-87 **Avg. Steps:** False
# of Steps: 4 **Avg. Test:** False
# of Loops: 0
Use Path: False **Result Path:**

Step#	Spd (rpm)	Inc. Spd	Use Tmp	Tmp (C°)	Inc. Tmp	Data Type	Data intr (sec)	Avr Dur (sec)	Point@ End	End Type	End Value	End Tol.	Density (g/cm3)	QCtype	QCL	QCH	In Test Avr.
1	20	False	False	40	False	Single	0	0	False	Time	90	0	0.00	None	0.00 %	0.00 %	False
2	25	False	False	40	False	Single	0	0	False	Time	90	0	0.00	None	0.00 %	0.00 %	False
3	30	False	False	40	False	Single	0	0	False	Time	90	0	0.00	None	0.00 %	0.00 %	False
4	35	False	False	40	False	Single	0	0	False	Time	90	0	0.00	None	0.00 %	0.00 %	False

Test Data:

Data Point	Step Point	Speed RPM	Torque (%)	Temperature °C	Time (Sec)	Viscosity cP	Shear Stress N/m²	Shear Rate 1/s
1	1	20.00	34.0	40.0	00:03:23.2	10.29	1.87	24.46
2	2	25.00	42.0	40.0	00:10:23.0	10.22	1.93	30.58
3	3	30.00	50.1	40.0	00:13:30.3	10.14	1.99	36.69
4	4	35.00	56.7	40.0	00:15:30.8	10.08	2.04	42.82

Data Report

Sample Information:
Sample Name: {pure 50C}

Tester Name: Salem Alhamd **Torque Coefficient:** 0.0937300026 **Result Notes:**
Run Time: 04/12/2022
Instr. S/N: 0
Torque Range: LV

Test Method:
Test Name {pure 50C}

Saved On: 04/12/2022 10:35:00 AM **Math Model:** None **Instruction** **Instruction**
Spindle: DIN-87 **Avg. Steps:** False
# of Steps: 4 **Avg. Test:** False
# of Loops: 0
Use Path: False **Result Path:**

Step#	Spd (rpm)	Inc. Spd	Use Tmp	Tmp (C°)	Inc. Tmp	Data Type	Data intr (sec)	Avr Dur (sec)	Point@ End	End Type	End Value	End Tol.	Density (g/cm3)	QCtype	QCL		QCH		In Test Avr.
1	20	True	False	50	False	Single	0	0	False	Time	60	0	0.00	None	0.00	%	0.00	%	False
2	25	False	False	50	False	Single	0	0	False	Time	60	0	0.00	None	0.00	%	0.00	%	False
3	30	False	False	50	False	Single	0	0	False	Time	60	0	0.00	None	0.00	%	0.00	%	False
4	35	False	False	50	False	Single	0	0	False	Time	60	0	0.00	None	0.00	%	0.00	%	False

Test Data:

Data Point	Step Point	Speed RPM	Torque (%)	Temperature °C	Time (Sec)	Viscosity cP	Shear Stress N/m²	Shear Rate 1/s
1	1	20.00	34.0	50.0	00:02:26.7	18.18	3.45	24.46
2	2	25.00	42.3	50.0	00:04:14.7	18.15	3.53	30.58
3	3	30.00	50.7	50.0	00:05:47.6	18.12	3.62	36.69
4	4	35.00	58.8	50.0	00:07:54.2	18.10	3.71	42.82

Rheocalc T V1.0.1

Brookfield Engineering Labs INC.

Data Report

Sample Information: **Sample Name:** {SM1 50C}

Tester Name: Salem Alhamd **Torque Coefficient:** 0.0937300026 **Result Notes:**

Run Time: 04/12/2022

Instr. S/N: 0

Torque Range: LV

Test Method: **Test Name** {SM1 50C}

Saved On: 04/12/2022 12:02:00 AM **Math Model:** None **Instruction** **Instruction**

Spindle: DIN-87 **Avg. Steps:** False

# of Steps: 4 **Avg. Test:** False

# of Loops: 0

Use Path: False **Result Path:**

Step#	Spd (rpm)	Inc. Spd	Use Tmp	Tmp (C°)	Inc. Tmp	Data Type	Data intr (sec)	Avr Dur (sec)	Point@ End	End Type	End Value	End Tol.	Density (g/cm3)	QCtype	QCL	QCH	In Test Avr.
1	20	False	False	50	False	Single	0	0	False	Time	90	0	0.00	None	0.00 %	0.00 %	False
2	25	False	False	50	False	Single	0	0	False	Time	90	0	0.00	None	0.00 %	0.00 %	False
3	30	False	False	50	False	Single	0	0	False	Time	90	0	0.00	None	0.00 %	0.00 %	False
4	35	False	False	50	False	Single	0	0	False	Time	90	0	0.00	None	0.00 %	0.00 %	False

Test Data:

Data Point	Step Point	Speed RPM	Torque (%)	Temperature °C	Time (Sec)	Viscosity cP	Shear Stress N/m²	Shear Rate 1/s
1	1	20.00	27.4	50.0	00:02:21.2	08.58	1.00	24.46
2	2	25.00	34.1	50.0	00:03:40.0	08.45	1.06	30.58
3	3	30.00	40.8	50.0	00:04:48.0	08.34	1.12	36.69
4	4	35.00	47.3	50.0	00:05:54.0	08.25	1.18	42.82

Data Report

Sample Information: **Sample Name:** {SM2 50C}

Tester Name: Salem Alhamd **Torque Coefficient:** 0.0937300026 **Result Notes:**
Run Time: 04/12/2022
Instr. S/N: 0
Torque Range: LV

Test Method: **Test Name** {SM2 50C}

Saved On: 04/12/2022 12:16:00 AM **Math Model:** None **Instruction** **Instruction**
Spindle: DIN-87 **Avg. Steps:** False
# of Steps: 4 **Avg. Test:** False
# of Loops: 0
Use Path: False **Result Path:**

Step#	Spd (rpm)	Inc. Spd	Use Tmp	Tmp (C°)	Inc. Tmp	Data Type	Data intr (sec)	Avr Dur (sec)	Point@ End	End Type	End Value	End Tol.	Density (g/cm3)	QCtype	QCL		QCH		In Test Avr.
1	20	False	False	50	False	Single	0	0	False	Time	90	0	0.00	None	0.00	%	0.00	%	False
2	25	False	False	50	False	Single	0	0	False	Time	90	0	0.00	None	0.00	%	0.00	%	False
3	30	False	False	50	False	Single	0	0	False	Time	90	0	0.00	None	0.00	%	0.00	%	False
4	35	False	False	50	False	Single	0	0	False	Time	90	0	0.00	None	0.00	%	0.00	%	False

Test Data:

Data Point	Step Point	Speed RPM	Torque (%)	Temperature °C	Time (Sec)	Viscosity cP	Shear Stress N/m²	Shear Rate 1/s
1	1	20.00	27.2	50.0	00:02:51.4	08.35	0.99	24.46
2	2	25.00	33.9	50.0	00:04:14.3	08.22	1.05	30.58
3	3	30.00	40.6	50.0	00:05:35.3	08.10	1.11	36.69
4	4	35.00	47.4	50.0	00:07:14.8	07.97	1.17	42.82

Rheocalc T V1.0.1

Brookfield Engineering Labs INC.

Data Report

Sample Information: **Sample Name:** {SM3 50C}

Tester Name: Salem Alhamd **Torque Coefficient:** 0.0937300026 **Result Notes:**

Run Time: 04/12/2022

Instr. S/N: 0

Torque Range: LV

Test Method: **Test Name** {SM3 50C}

Saved On: 04/12/2022 12:35:00 AM **Math Model:** None **Instruction** **Instruction**

Spindle: DIN-87 **Avg. Steps:** False

# of Steps: 4 **Avg. Test:** False

# of Loops: 0

Use Path: False **Result Path:**

Step#	Spd (rpm)	Inc. Spd	Use Tmp	Tmp (C°)	Inc. Tmp	Data Type	Data intr (sec)	Avr Dur (sec)	Point@ End	End Type	End Value	End Tol.	Density (g/cm3)	QCtype	QCL		QCH		In Test Avr.
1	20	False	False	50	False	Single	0	0	False	Time	90	0	0.00	None	0.00	%	0.00	%	False
2	25	False	False	50	False	Single	0	0	False	Time	90	0	0.00	None	0.00	%	0.00	%	False
3	30	False	False	50	False	Single	0	0	False	Time	90	0	0.00	None	0.00	%	0.00	%	False
4	35	False	False	50	False	Single	0	0	False	Time	90	0	0.00	None	0.00	%	0.00	%	False

Test Data:

Data Point	Step Point	Speed RPM	Torque (%)	Temperature °C	Time (Sec)	Viscosity cP	Shear Stress N/m²	Shear Rate 1/s
1	1	20.00	26.1	50.0	00:02:56.0	08.14	0.98	24.46
2	2	25.00	32.6	50.0	00:04:16.4	07.99	1.04	30.58
3	3	30.00	39.2	50.0	00:05:19.1	07.90	1.10	36.69
4	4	35.00	46.2	50.0	00:05:52.6	07.82	1.15	42.82

Rheocalc T V1.0.1
Brookfield Engineering Labs INC.

Data Report

Sample Information:

Sample Name: {SM4 50C}

Tester Name: Salem Alhamd **Torque Coefficient:** 0.0937300026 **Result Notes:**
Run Time: 04/12/2022
Instr. S/N: 0
Torque Range: LV

Test Method:

Test Name {SM4 50C}

Saved On: 04/12/2022 12:50:00 AM **Math Model:** None **Instruction** **Instruction**
Spindle: DIN-87 **Avg. Steps:** False
# of Steps: 4 **Avg. Test:** False
# of Loops: 0
Use Path: False **Result Path:**

Step#	Spd (rpm)	Inc. Spd	Use Tmp	Tmp (C°)	Inc. Tmp	Data Type	Data intr (sec)	Avr Dur (sec)	Point@ End	End Type	End Value	End Tol.	Density (g/cm3)	QCtype	QCL	QCH	In Test Avr.
1	20	False	False	50	False	Single	0	0	False	Time	90	0	0.00	None	0.00 %	0.00 %	False
2	25	False	False	50	False	Single	0	0	False	Time	90	0	0.00	None	0.00 %	0.00 %	False
3	30	False	False	50	False	Single	0	0	False	Time	90	0	0.00	None	0.00 %	0.00 %	False
4	35	False	False	50	False	Single	0	0	False	Time	90	0	0.00	None	0.00 %	0.00 %	False

Test Data:

Data Point	Step Point	Speed RPM	Torque (%)	Temperature °C	Time (Sec)	Viscosity cP	Shear Stress N/m²	Shear Rate 1/s
1	1	20.00	26.8	50.0	00:00:09.7	07.92	0.97	24.46
2	2	25.00	33.2	50.0	00:00:46.9	07.87	1.03	30.58
3	3	30.00	40.0	50.0	00:01:41.4	07.84	1.09	36.69
4	4	35.00	46.1	50.0	00:02:28.8	07.80	1.14	42.82
